# Special Issue “The Prevention, Treatment, and Complications of Diabetes Mellitus”

**DOI:** 10.3390/jcm11185305

**Published:** 2022-09-09

**Authors:** Ana I. Arroba, Manuel Aguilar-Diosdado

**Affiliations:** 1 Research Unit, Biomedical Research and Innovation Institute of Cadiz (INiBICA), 11009 Cádiz, Spain; 2 Department of Endocrinology and Metabolism, Hospital Puerta del Mar, 11009 Cádiz, Spain; 3 School of Medicine, Cadiz University (UCA), Ana de Viya 21, 11009 Cadiz, Spain

Diabetes mellitus (DM) is a world health problem of global repercussion. It is expected that, in the next 20 years, the number of patients with DM will increase to 642 million people [1]. Type 1 diabetes mellitus (T1DM) responds to a multifactorial pathogenesis essentially linked to an autoimmune aggression mediated by T cells and autoantibodies that generate a progressive loss of insulin producing cells in the pancreas [2]. On the other hand, the pathogenesis of type 2 diabetes mellitus (T2DM) is essentially linked to the development of a state of resistance to the actions of insulin [3]. Both types are the consequence of an interaction of environmental, epigenetic, and genetic factors [4]. Genetic factors promote a special susceptibility to the development of the disease and epigenetics is considered as the link between the environment and genetics, altering gene and protein expression that could affect in autoimmunity and in the vulnerability of beta cells of pancreatic islets to the external factors.

In fact, clinical practice guidelines state that good metabolic—glycemic—control contributes to the reduction of complications associated with T1DM [5] and with T2DM [6] and that the best parameter to define the degree of glycemic control is glycosylated hemoglobin (HbA1c) which provides the average blood glucose levels in the last 2–3 months [5,6]. In addition, the development of new technologies to improve the management of DM, such as continuous glucose monitoring systems (CGMS) that measure glucose in interstitial fluid, show that they are the best way to monitor glucose levels to avoid hypoglycemia and to reduce glucose excursions. In fact, different studies have shown that these devices significantly reduce the number and intensity of hypoglycemia and improve HbA1c levels in both T1DM [7] and T2DM patients receiving insulin therapy [8].

However, the main challenge to clinicians is to reduce morbidity and mortality linked to DM, since complications associated with DM progression can arise both in the short and long term of the disease evolution. Hyperglycemia is a frequent finding in both hospitalized [9] and outpatients [10] and the management of glucose levels is the target on the most clinical trials. The use of modern insulin pumps offer a great variety of possibilities to which is added the incorporation of sensors-augmented insulin-pump, thus allowing its regulation and a higher and better level of safety in the device [11]. In T2DM, the treatment with non-insulin agents [12] contributes to improve the efficiency of insulin on glycemia levels and reflects the need to find a specific and personalized therapies for the profile of each patient. Actually, there is an increasing need to identify and provide evidence about the efficiency and safety of new therapy modalities and some of them have been included in this Special Issue.

DM is a life-threatening disease that causes complications and is considered as a serious disorder that doubles the risk of premature death [13]. Some aspects related with different fields into the DM progression have also been addressed in this Special Issue, such as the obstructive sleep apnea due to hypoxia implications [14], microvascular [15] and macrovascular [16] complications, elevated low-density lipoprotein (LDL) and cholesterol [17], and different oral processes [18]. A specifically complication associated with gestational diabetes mellitus (GDM) is the increased risk of hypertensive disorders; in this regard, new models based on biomarker parameters allow us to detect patients with GDM and these disorders and start earlier preventive strategies [19].

Upon the improvement of DM management, the scientific knowledge of the different underlying processes is very important. The implication of molecular mechanisms that could justify an adequate treatment option or the optimal technologies for an early diagnosis have to be included into the structural organigram of the study and follow-up of DM. The potential new biochemical targets, such as IL-1 in T1DM [20] or insulin receptor in T2DM [21], involve a deep analysis of the intracellular signaling and its potential implication in the physiopathology of the disease.

The prevention of complications of DM is relevant and requires an early diagnosis together with adequate treatment and follow-up of the patient. The most effective preventive strategy to avoid and/or delay the onset and development of DM complications is early diagnosis and early intervention aimed at mitigating symptoms and reducing sequelae and costs.
